# Effect and Mechanisms of Quercetin for Experimental Focal Cerebral Ischemia: A Systematic Review and Meta-Analysis

**DOI:** 10.1155/2022/9749461

**Published:** 2022-02-25

**Authors:** Chao Guo, Wen-Jun Wang, Yu-Cheng Liao, Chao Zhao, Ying Yin, Min-Na Yao, Yi Ding, Jing-Wen Wang

**Affiliations:** Department of Pharmacy, Xijing Hospital, Fourth Military Medical University, Xi'an, China

## Abstract

Quercetin, a naturally occurring flavonoid, is mainly extracted from tea, onions, and apples. It has the underlying neuroprotective effect on experimental ischemic stroke. A systematic review and meta-analysis were used to assess quercetin's efficacy and possible mechanisms in treating focal cerebral ischemia. Compared with the control group, twelve studies reported a remarkable function of quercetin in improving the neurological function score (NFS) (*P* < 0.05), and twelve studies reported a significant effect on reducing infarct volume (*P* < 0.05). Moreover, two and three studies showed that quercetin could alleviate blood-brain barrier (BBB) permeability and brain water content, respectively. The mechanisms of quercetin against focal cerebral ischemia are diverse, involving antioxidation, antiapoptotic, anti-inflammation, and calcium overload reduction. On the whole, the present study suggested that quercetin can exert a protective effect on experimental ischemic stroke. Although the effect size may be overestimated because of the quality of studies and possible publication bias, these results indicated that quercetin might be a promising neuroprotective agent for human ischemic stroke. This study is registered with PROSPERO, number CRD 42021275656.

## 1. Introduction

Stroke is recognized as one of the significant causes of death and disability, of which ischemic stroke is the primary type [1]. According to the World Health Organization (WHO) report, about 15 million people worldwide suffer strokes each year [2]. Pharmacologic thrombolysis and endovascular thrombectomy are currently available treatments for ischemic stroke. However, both treatments in clinical usage are limited due to the strict timing criteria and contraindications, and their benefits diminish with delays in therapy initiation [3]. Therefore, existing ischemic stroke treatment methods must be reformed, and new pharmacological treatment modalities should be explored.

**Figure 1:**
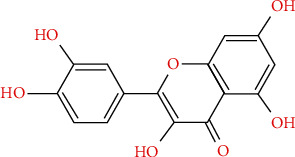
Chemical structures of quercetin.

Flavonoids are widely found in plant and vegetable diets. They are reported to have antiviral, anti-inflammatory, heart protection, antidiabetes, anticancer, antiaging, and other biological activities [4–9]. Quercetin (Figure 1), the predominant dietary flavonoid, is mainly found in tea, onions, and apples. A previous review showed that quercetin protects cells against oxidative stress damage in various organs during ischemia-reperfusion (I/R) [10]. Quercetin can improve blood-brain barrier dysfunction [11] and reduce neuronal apoptosis after cerebral I/R injury [12]. The randomized, double-blind, placebo-controlled clinical trial reported that quercetin could decrease ambulatory blood pressure (ABP) in patients with hypertension [13], indicating that it has the effect of protecting cardiovascular and cerebrovascular diseases. Another clinical trial also revealed that quercetin possesses a protective effect on central hemodynamic parameters and myocardial ischemia in patients with stable coronary heart disease [14]. Furthermore, the oral clearance of quercetin is high, with an average terminal half-life of 3.5 h in healthy humans [15], suggesting that it may be a candidate drug for clinical application. These studies indicated that quercetin has an excellent therapeutic effect on cardiovascular and cerebrovascular diseases such as ischemic stroke, which should be supported by the pooling data from preclinical studies.

The systematic review is a type of secondary research that gathers all primary research that meets prespecified qualification criteria for solving a specific research problem, minimizing bias [16]. It can provide convincing evidence and help choose the optimal drug administration requirements in clinical trials. However, to date, there has not been a systematic review to investigate the compliance of experimental studies of quercetin on ischemic stroke models. Here, we conducted a preclinical systematic review to appraise the effectiveness and mechanism of action of quercetin in the treatment of ischemic stroke in animal models.

## 2. Methods

This systematic review and meta-analysis were carried out according to the methods of Wang et al. [17].

### 2.1. Database and Search Strategy

The following databases were searched: PubMed, Web of Science, Chinese Biomedical Literature Database (SinoMed), China National Knowledge Infrastructure (CNKI), WanFang Database, and VIP Database. We collected all studies from inception to Aug 2021. Our search terms were as follows: “Quercetin AND ischemic(a) stroke OR cerebral ischemic(a) injury OR cerebral ischemic(a) reperfusion OR cerebral infarct OR middle cerebral artery occlusion (MCAO).” There are no restrictions on the country or language of publication.

### 2.2. Inclusion Criteria

All animal experiments assessing the effect of quercetin on focal cerebral ischemia were chosen, regardless of animal species, age, and sex. The following screening criteria should be satisfied: (1) quercetin was administered to an animal model of focal cerebral ischemia, regardless of the dosage, route, method, and treatment schedule; (2) animal models of focal cerebral ischemia involved temporary or permanent middle cerebral artery occlusion (MCAO); (3) the intervention group only used quercetin treatment; and (4) control animals received no treatment or vehicle. The primary outcome indicators included NFS and infarct volume; the second outcome indicators were BBB permeability and brain water content.

### 2.3. Exclusion Criteria

The following were exclusion criteria: (1) the study was a viewpoint, review, case report, abstract, in vitro experiment, ex vivo study, or human study; (2) nonfocal cerebral ischemia models such as chronic cerebral ischemia, global cerebral ischemia, traumatic models, or hypoxia-ischemia; (3) non-quercetin-based interventions, quercetin modifications, and combinations with other compounds and treatments; (4) no control group; and (5) no statement of sample size.

### 2.4. Data Extraction

The detailed information from included studies was extracted by two independent reviewers, as outlined below: (1) first author, publication year, focal cerebral ischemia model, and anesthesia method; (2) animal species, sex, and weight; (3) the dose, strategy, and frequency of administration of quercetin and the control groups; (4) the data of mean value and standard deviation of NFS, infarct volume, BBB permeability, and/or brain water content; and (5) the timing and sample size for outcome assessments were also extracted. The highest dose data were extracted when the treatment group contained various dose subgroups. If data are derived from different time points, the result at the peak time point is extracted. When data is only displayed as a graph, we contact the author for detailed information. If no response is received, we will use the graphic digitizer software to measure the value or exclude it.

### 2.5. Quality Evaluation

Two independent reviewers assessed the quality of included studies according to a 10-item modified checklist [18]: (1) published in a peer-reviewed journal; (2) controlled temperature; (3) randomized treatment or control; (4) blinded evaluation of outcome; (5) avoidance of intrinsically neuroprotective anesthetics; (6) animal and/or model (aged, diabetic, or hypertensive); (7) sample size calculation; (8) compliance with animal welfare regulations; (9) reporting potential conflicts of interest; and (10) injury confirmed via the laser Doppler or perfusion imaging.

### 2.6. Statistical Analysis

The Review Manager (version 5.3) software was used for statistical analysis. The estimate of the pooled effect sizes is calculated by the standardized mean difference (SMD) using a fixed-effects model without statistical evidence of heterogeneity (*P* ≥ 0.1, *I*^2^ ≤ 50%). The random-effects (RE) model is applied with statistical heterogeneity (*P* < 0.1, *I*^2^ > 50%). The statistical significance is *P* < 0.05, and the 95% confidence interval for all results is computed. The publication bias was evaluated by the funnel plot and the Egger test.

## 3. Results

### 3.1. Study Selection

**Figure 2:**
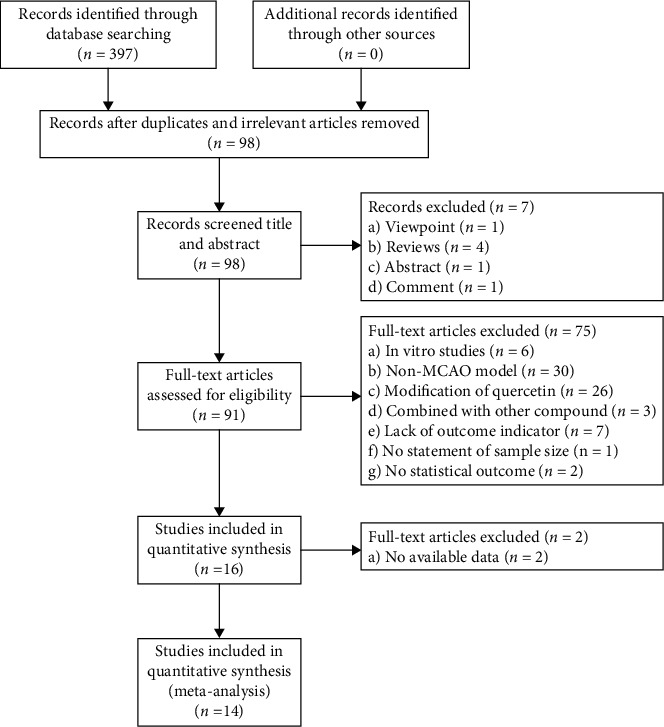
Flow diagram of the search process.

A total of 397 studies were found through database search, and 98 unique studies were identified after removing 299 duplicate and irrelevant studies. After screening the titles and abstracts, 7 studies were excluded for at least one of the following reasons: opinions, comments, and abstracts. The remaining 91 studies were read in detail, of which 75 studies were removed subsequently for the following reasons: (1) in vitro studies; (2) not focal cerebral ischemia model; (3) administration of modification of quercetin; (4) combination with other compounds; (5) lack of outcome indicators; (6) no statement of sample size; and (7) no statistical outcome. Finally, 14 studies were selected for quantitative analysis after excluding two articles for which no available data can be obtained.  Figure 2 shows the screening process.

### 3.2. Features of Included Studies

Between 2011 and 2021, twelve studies were published in English, and two were published in Chinese. The fourteen studies included Wistar rats [19, 20] and Sprague-Dawley rats [21–32]. The weight of rat was between 180 and 320 g. All studies used male animals, and no studies used aged, diabetic, or hypertensive animals. For anesthesia, six studies used Zoletil [24, 25, 27, 29, 30, 32], four studies used chloral hydrate [19, 22, 23, 26], two studies used isoflurane [20, 28], one study used pentobarbital sodium [31], and one study used ketamine [21]. Cerebral ischemic injury was simulated by temporary middle cerebral artery occlusion (tMCAO) in eight studies [19–23, 26, 28, 31] and permanent MCAO (pMCAO) in six studies [24, 25, 27, 29, 30, 32]. Seven studies described tMCAO ischemia times ranging from 1 to 2 hours, while one study did not mention it [31]. The ischemic time of pMCAO in six studies was 24 hours. Four studies carried out a dose gradient of quercetin [20, 22, 26, 31]. Among them, two studies implemented 25, 50, and 100 mg/kg [26, 31], one study utilized 10 and 20 mg/kg [22], and the other adopted 10, 30, and 50 mg/kg [20]. Ten studies performed single dose, in which six of them used 10 mg/kg [21, 24, 27, 29, 30, 32], two studies used 30 mg/kg [19, 25], and the others used 7.5 [23] and 25 mg/kg [28], respectively. Ten studies [21, 24–32] administrated quercetin before ischemia, three [20, 22, 23] administrated quercetin after ischemia, and one [19] gave quercetin before ischemia and after reperfusion. Quercetin was administered by intraperitoneal injection (IP) in 11 studies and intragastric (IG) in 3 studies [22, 26, 31]. NSF was reported in 12 studies [20–28, 30–32], infarct volume in 12 studies [19–25, 27–31], brain water content in 3 studies [25, 26, 29], and BBB permeability in 2 studies [20, 28]. More details about the features of these studies are shown in Table 1.

### 3.3. Study Quality

A 10-point scoring method was used to evaluate the quality of studies (Table 2). The study quality score ranged from 3 to 6 points, with an average of 4.5 points. All included studies were peer-reviewed publications. Nine studies stated random allocation to control or treatment and temperature control. Three described blinding their outcome assessment. Twelve studies used anesthetics without significant intrinsic neuroprotective activity. None of the studies used animals or models with relevant comorbidities and the sample size calculation. Nine studies reported compliances with animal welfare regulations, and six showed no potential conflict of interests; only one study describing the MCAO model was confirmed via the laser Doppler flow analyzer.

### 3.4. Effectiveness Assessment

#### 3.4.1. NFS

**Figure 3:**
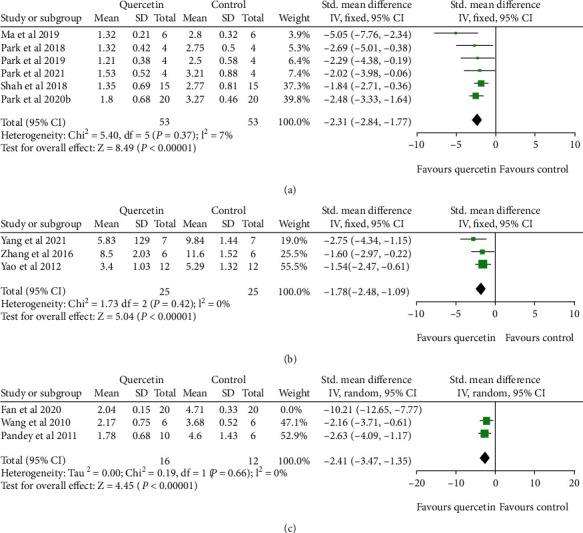
The forest plots: the effects of quercetin for improving NFS compared with the control group according to the (a) Bederson criterion, (b) mNSS standard, and (c) Longa criteria.

Meta-analysis of six studies [24–27, 30, 32] revealed that quercetin has a prominent effect in improving NFS compared with the control group according to the Bederson criterion (*n*_Q_/*n*_C_ = 53/53, SMD: -2.31, 95% CI [-2.84, -1.77], *P* < 0.00001; heterogeneity: Chi^2^ = 5.40, df = 5 (*P* = 0.37); *I*^2^ = 7%) (Figure 3(a)). A meta-analysis of three studies [20, 22, 23] showed a remarkable effect of quercetin for improving NFS based on the mNSS standard (*n*_Q_/*n*_C_ = 25/25, SMD: -1.78, 95% CI [-2.48, -1.09], *P* < 0.00001; heterogeneity: Chi^2^ = 1.73, df = 2 (*P* = 0.42); *I*^2^ = 0%) (Figure 3(b)). In addition, three studies [21, 28, 31] also revealed that quercetin significantly improved the NFS according to the Longa criteria (*n*_Q_/*n*_C_ = 36/32, SMD: -4.88, 95% CI [-9.01, -0.75], *P* = 0.02; heterogeneity: Chi^2^ = 33.29, df = 2 (*P* = 0.001); *I*^2^ = 94%). One study [31] was removed due to its outlier data resulting in heterogeneity. The meta-analysis of the two residual studies showed that quercetin could promote recovery of neurological function (*n*_Q_/*n*_C_ = 16/12, SMD: -2.41, 95% CI [-3.47, -1.35], *P* < 0.00001; heterogeneity: Chi^2^ = 0.19, df = 1 (*P* = 0.66); *I*^2^ = 0%) (Figure 3(c)).

#### 3.4.2. Infarct Volume

**Figure 4:**
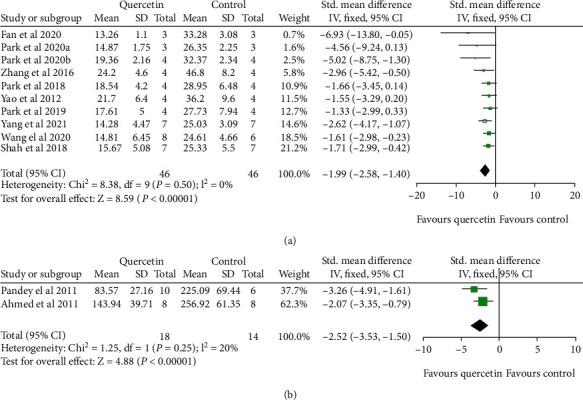
The forest plots: the effects of quercetin for reducing infarct volume compared with the control group (MCAO) according to (a) percentage calculation and (b) mm^3^.

According to the percentage calculation, a meta-analysis of ten studies [20, 22–25, 27–31] showed that quercetin significantly reduces infarct volume in the MCAO model compared with the control group (*n*_Q_/*n*_C_ = 46/46, SMD: -1.99, 95% CI [-2.58, -1.40], *P* < 0.00001; heterogeneity: Chi^2^ = 8.38, df = 9 (*P* = 0.50); *I*^2^ = 0%) (Figure 4(a)). Two studies [19, 21] reported infarct volume (mm^3^) as the outcome measurement, and their meta-analysis showed that quercetin has a significant effect on reducing infarct volume during cerebral ischemic injury (*n*_Q_/*n*_C_ = 18/14, SMD: -2.52, 95% CI [-3.53, -1.50], *P* < 0.00001; heterogeneity: Chi^2^ = 1.25, df = 1 (*P* = 0.25); *I*^2^ = 20%) (Figure 4(b)).

#### 3.4.3. Brain Content Water

**Figure 5:**
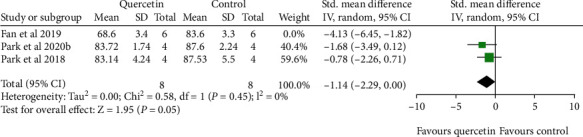
The forest plots: the effects of quercetin for decreasing brain water content compared with the control group (MCAO).

Meta-analysis of three studies revealed a significant effect of quercetin for decreasing brain content water (*n*_Q_/*n*_C_ = 14/14, SMD: -2.02, 95% CI [-3.82, -0.22], *P* = 0.03; heterogeneity: Tau^2^ = 1.63, Chi^2^ = 5.73, df = 2 (*P* = 0.06); 𝐼^2^ = 65%). Because of considerable heterogeneity, we deleted the outlier study. The meta-analysis of the remaining two studies showed there is no heterogeneity (*n*_Q_/*n*_C_ = 8/8, SMD: -1.14, 95% CI [-2.29, 0.00], *P* = 0.05; heterogeneity: Tau^2^ = 0.00, Chi^2^ = 0.58, df = 1 (*P* = 0.45); 𝐼^2^ = 0%) (Figure 5).

#### 3.4.4. BBB Permeability

Two studies reported BBB permeability based on the Evans blue assay. It failed for pool analysis because one study measured the content of EB in the ischemic hemisphere, and another measured Evans blue's content in the ischemic cortex. However, they all confirmed the outstanding effects of quercetin for meliorating the BBB permeability induced by focal cerebral ischemia (*P* < 0.05).

#### 3.4.5. Publication Bias

**Figure 6:**
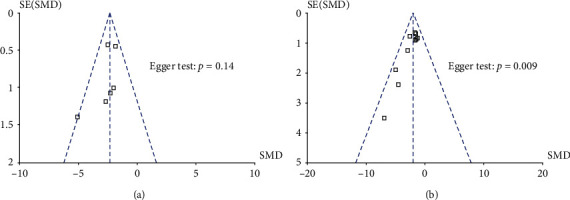
Publication bias for (a) NFS and (b) infarct volume.

The Egger test and funnel plot were used to assess publication bias for NFS (Bederson criterion) and infarct volume (%). As shown in Figure 6, there was no significant publication bias in NFS (*P* = 0.14), whereas there may have publication bias in infarct volume (*P* = 0.009).

## 4. Discussion

### 4.1. Summary of Evidence

A total of fourteen studies were included, and the period of included studies is from 2011 to 2021. The main findings of the present systematic review showed that quercetin could improve NFS, reduce the infarct volume, and protect BBB integrity during focal cerebral ischaemia, suggesting that quercetin exerted the neuroprotection for acute ischemic stroke. The neuroprotective mechanisms of quercetin are mainly mediated by its antioxidant, anti-inflammatory, antiapoptosis properties, and resistance to calcium overload.

### 4.2. Strengths and Limitations

The strength of the present study is that this is the first meta-analysis to evaluate the effects of quercetin against focal cerebral ischemia. In this meta-analysis, we use six databases with many terms and keywords to increase the number of searches and ensure the extensive retrieval of published articles. Moreover, we clearly defined animal models and outcome indicators before the meta-analysis, which reduced bias in selecting the included studies. Our results provide the practical value for the evidence-based transformation of animal data from the laboratory to the bedside.

The present study also has some limitations. First of all, all the databases we searched are in English or Chinese, which might lead to selection bias because studies published in other languages may be excluded. Second, no studies reported the negative effect of quercetin on infarct volume, which may be a pivotal contributor to publication bias, as positive results and large sample sizes are always easier to publish than negative results and small sample sizes. The effective method for avoiding publication bias is to include unpublished studies and trial registries [33]. Third, the methodological quality of the included studies is average. The included studies lacked blinded assessments, no sample size calculations, animals without relevant comorbidities, and no testing on successfully modeled animals. Therefore, some conclusions in the present study should be referenced critically.

### 4.3. Implications

High-quality methodologies of studies are the elements of drugs translating from animal research into clinical trials for human disease [34]. The methodological quality of these studies was moderate, especially none of the included studies estimated sample size. Insufficient sample size can blunt the actual effect of intervention in the experiment, while the vast sample size can lead to animal waste and induce ethical concerns about animals. Only three studies were blinded to evaluate the experiment results, resulting in overestimating of treatment effects of quercetin. Animals with relevant comorbidities were not used in these studies, which do not truly represent human pathology under clinical conditions. Animal Research Reporting of In Vivo Experiments (ARRIVE) [35] with a 20-item list of introductions is recommended as the criterion for further studies on quercetin to treat ischemic stroke, which might considerably improve the quality of the methodology.

### 4.4. The Primary Mechanism of Quercetin for Ischemic Stroke

The neuroprotective mechanisms of quercetin against ischemic stroke are summarized as follows: (1) Oxidative stress plays a vital role in the pathogenesis of ischemic stroke [36]. The excessive reactive oxygen species (ROS) induce oxidative stress during cerebral ischemia injury [37]. Quercetin could reduce oxidative stress [19, 20, 26, 28, 31] by decreasing the content of MDA [26, 28, 31], heightening the activity of SOD [19, 26, 31] and GSH [19]. These studies suggested that quercetin can significantly inhibit oxidative stress and reduce the neurotoxicity of free radicals. (2) Cytokines are the main factors regulating the inflammatory response during cerebral ischemic injury [38]. Quercetin could inhibit inflammatory response by downregulating the expression of proinflammatory cytokines including TNF-*α*, IL-1*β*, and IL-6 [23, 28] and upregulating the expression of anti-inflammatory cytokines including IL-4 and IL-10 [23]. (3) Apoptosis is a type of cell death under ischemia conditions [39]. Quercetin exerts antiapoptotic effects through downregulating PARP and caspase-3 expression [19, 22, 25, 28] and inhibiting the reduction of thioredoxin and apoptosis signal-regulating kinase 1 (ASK1) binding [29]. (4) Calcium overload leads to neuronal cell death and brain damage after cerebral ischemia [40]. Parvalbumin and hippocalcin are the calcium-buffering proteins that play vital roles in reducing calcium overload in glutamate-exposed neuronal cells [41]. Quercetin attenuates calcium overload by preventing the reduction of parvalbumin and hippocalcin expression [30, 32] and decreasing calpain-mediated SBDP [21]. (5) Quercetin alleviates abnormal autophagy via inhibiting the signaling pathway of AMPK/mTOR/ULK1 [26]. (6) Quercetin protects the integrity of BBB via the Sirt pathway [20]. (7) Quercetin attenuates glutamate toxicity and improves cellular dysfunction [24, 27, 29]. (8) Quercetin enhances ERK/Akt phosphorylation11 and activates BDNF-TrkB-PI3K/Akt [22]. (9) Quercetin improves energy metabolism disorder by upregulating the activity of Na+-K+-ATPase [19]. (10) Quercetin promotes axonal outgrowth, neuronal differentiation, and neurogenesis [24, 27]. Altogether, these findings indicate that quercetin would be a promising therapeutic and protective agent against ischemic stroke.

## 5. Conclusion

Quercetin can reduce cerebral infarction volume, nerve function deficit, BBB permeability, and brain edema and protect cerebral ischemia injury through various signaling pathways. Other undisclosed molecular mechanisms deserve further study.

## Figures and Tables

**Table 1:** Characteristics of 14 included studies.

Author	Species (sex)	Weight	Model	Anesthetic	Treatment	Control	Outcome index	Intergroup differences
Ahmad et al. 2011	Male Wistar rats	250-300 g	tMCAO for 2 h	Chloral hydrate (400 mg/kg, IP)	Quercetin, 30 mg/kg, IP, at 1 h before MCAO and then 0, 24, 48, and 72 h after MCAO	The same volume of 0.1% DMSO, IP, at 30 min before MCAO and then 0, 24, 48, and 72 h after MCAO	(1) Infarct volume (TTC), 72 h after MCAO (8/8)	*P* < 0.05
							(2) TBARS level, 72 h after MCAO (8/8)	*P* < 0.05
							(3) GSH content, 72 h after MCAO (8/8)	*P* < 0.05
							(4) Activities of antioxidant enzymes, 72 h after MCAO (8/8)	*P* < 0.05
							(5) Activity of Na^+^-K^+^-ATPase, 72 h after MCAO (8/8)	*P* < 0.05
							(6) PARP activity, 72 h after MCAO (8/8)	*P* < 0.05
							(7) Activity of caspase-3, 72 h after MCAO (8/8)	*P* < 0.05
							(8) Number of p53 positive cells, 72 h after MCAO (8/8)	*P* < 0.05
Pandey et al. 2011	Male SD rats	240-260 g	tMCAO for 1 h	Ketamine (50 mg/kg IP)	Quercetin, 10 mg/kg, IP, at 30 min before MCAO	The same volume of normal saline, IP, at 30 min before MCAO	(1) NFS (Longa), 24 h after MCAO (6/10)	*P* < 0.05
							(2) Infarct volume (TTC), 24 h after MCAO (6/10)	*P* < 0.05
							(3) Nitrite levels, 20 min after MCAO (6/6)	*P* < 0.05
							(4) MDA levels, 20 min after MCAO (6/6)	*P* < 0.05
							(5) Spectrin breakdown products (SBDPs) expression, 24 h after MCAO (6/6)	*P* < 0.01
Yao et al. 2012	Male SD rats	250-270	tMCAO for 1.5 h	10% chloral hydrate (0.4 ml/kg, IP)	Quercetin, 10, 20 mg/kg, IG, at 3 h after MCAO and then once daily	The same volume of 0.1% dH_2_O/0.1% tween-80, IG, at 3 h after MCAO and then once daily	(1) NFS (mNSS), 28 d after MCAO (12/12)	*P* < 0.05
							(2) Infarct volume, 7 d after MCAO (4/4)	*P* < 0.05
							(3) TUNEL-positive cells, 7 d after MCAO (4/4)	*P* < 0.01
							(4) Bcl-2 levels, 7 d after MCAO (4/4)	*P* < 0.01
							(5) Bax levels, 7 d after MCAO (4/4)	*P* < 0.01
							(6) Cleaved caspase-3/caspase-3, 7 d after MCAO (4/4)	*P* < 0.05
							(7) BDNF levels, 7 d after MCAO (4/4)	*P* < 0.01
							(8) TrkB levels, 7 d after MCAO (4/4)	*P* < 0.01
							(9) p-AKT/AKT levels, 7 d after MCAO (4/4)	*P* < 0.01
Zhang et al. 2016	Male SD rats	200-300 g	tMCAO for 2 h	10% chloral hydrate	Quercetin, 7.5 mg/kg, IP, at 1 h after MCAO and then every 12 h for 3 days	The same volume of 0.1% DMSO, IP, at 1 h after MCAO and then every 12 h for 3 days	(1) NFS (mNSS), 28 d after MCAO (6/6)	*P* < 0.05
							(2) Infarct volume (TTC), 28 d after MCAO (4/4)	*P* < 0.05
							(3) IL-6 levels, 14 d after MCAO (12/12)	*P* < 0.05
							(4) IL-1*β* levels, 14 d after MCAO (12/12)	*P* < 0.05
							(5) IL-4 levels, 14 d after MCAO (12/12)	*P* < 0.01
							(6) IL-10 levels, 14 d after MCAO (12/12)	*P* < 0.01
							(7) Caspase-3 immunoreactive staining, 28 d after MCAO (4/4)	*P* < 0.05
Shah et al. 2018	Male SD rats	200-230 g	pMCAO for 24 h	Zoletil (50 mg/kg, IM)	Quercetin, 10 mg/kg, IP, at 30 min before MCAO	The same volume of 0.05% DMSO, IP, at 30 min before MCAO	(1) NFS (Bederson), 24 h after MCAO (15/15)	*P* < 0.05
							(2) Infarct volume (TTC), 24 h after MCAO (7/7)	*P* < 0.05
							(3) ICDH and ICDH mRNA levels, 24 h after MCAO (4/4)	*P* < 0.05
							(4) Adenosylhomocysteinase and mRNA levels, 24 h after MCAO (4/4)	*P* < 0.05
							(5) Pyruvate kinase and mRNA levels, 24 h after MCAO (4/4)	*P* < 0.05
							(6) Ubiquitin carboxy-terminal hydrolase L1 (UCHL1) and UCHL1 mRNA levels, 24 h after MCAO (4/4)	*P* < 0.05
							(7) Heat shock protein 60 (HSP60) and HSP60 mRNA levels, 24 h after MCAO (4/4)	*P* < 0.05
							(8) Collapsin response mediator protein 2 (CRMP2) and CPM2 levels, 24 h after MCAO (4/4)	*P* < 0.05
Park et al. 2018	Male SD rats	200-220 g	pMCAO for 24 h	Zoletil (50 mg/kg, IM)	Quercetin, 30 mg/kg, IP, at 1 h before MCAO	The same volume of 0.05% DMSO, IP, at 1 h before MCAO	(1) NFS (Bederson), 24 h after MCAO (4/4)	*P* < 0.05
							(2) Infarct volume (TTC), 24 h after MCAO (4/4)	*P* < 0.05
							(3) Brain water content, 24 h after MCAO (4/4)	*P* < 0.05
							(4) Fluoro-Jade B staining, 24 h after MCAO (4/4)	*P* < 0.05
							(5) PARP levels, 24 h after MCAO (4/4)	*P* < 0.05
							(6) Caspase-3 levels, 24 h after MCAO (4/4)	*P* < 0.05
Ma et al. 2019	Male SD rats	180-220 g	tMCAO for 2 h	10% chloral hydrate	Quercetin, 25, 50, 100 mg/kg, IG, once daily for 14 days before MCAO	The same volume of 0.5% CMC-Na, IG, once daily for 14 days before MCAO	(1) NFS (Bederson), 24 h after MCAO (6/6)	*P* < 0.01
							(2) Brain water content, 24 h after MCAO (6/6)	*P* < 0.01
							(3) LDH/MDA/SOD, 24 h after MCAO (6/6)	*P* < 0.01
							(4) Beclin, 24 h after MCAO (6/6)	*P* < 0.01
							(5) LC3II/LC3I levels, 24 h after MCAO (6/6)	*P* < 0.01
							(6) p62/Bax/Bcl-2, 24 h after MCAO (6/6)	*P* < 0.01
							(7) p-AMPK, 24 h after MCAO (6/6)	*P* < 0.01
							(8) p-mTOR, 24 h after MCAO (6/6)	*P* < 0.01
							(9) p-ULK1, 24 h after MCAO (6/6)	*P* < 0.01
Park et al. 2019	Male SD rats	220-230 g	pMCAO for 24 h	Zoletil (50 mg/kg, IM)	Quercetin, 10 mg/kg, IP, at 30 min before MCAO	The same volume of 0.1% DMSO, IP, at 30 min before MCAO	(1) NFS (Bederson), 24 h after MCAO (4/4)	*P* < 0.05
							(2) Infarct volume (TTC), 24 h after MCAO (4/4)	*P* < 0.05
							(3) MALDI-TOF analysis for protein phosphatase 2A (PP2A) subunit B levels, 24 h after MCAO (4/4)	*P* < 0.05
							(4) RT-PCR analysis for PP2A subunit B levels, 24 h after MCAO (4/4)	*P* < 0.05
							(5) Western blot analysis for PP2A subunit B levels, 24 h after MCAO (4/4)	*P* < 0.05
Wang el al.2020	Male SD rats	250-300 g	tMCAO for 1.5 h	2–4% isoflurane	25 mg/kg, IP. Once daily for 21 days before MCAO	The same volume of saline vehicle, IP, once a day for 21 days before MCAO	(1) NFS (Longa), 72 h after MCAO (6/6)	*P* < 0.05
							(2) Infarct volume (TTC), 72 h after MCAO (6/6)	*P* < 0.05
							(3) BBB permeability (EB), 72 h after MCAO (6/6)	*P* < 0.05
							(4) Caspase 3 activity, 72 h after MCAO (6/6)	*P* < 0.05
							(5) MDA content, 72 h after MCAO (6/6)	*P* < 0.05
							(6) TNF-*α* and IL-1*β* mRNAs, 72 h after MCAO (6/6)	*P* < 0.05
							(7) p-ERK and p-AKT levels, 72 h after MCAO (6/6)	*P* < 0.05
Park et al. 2020a	Male SD rats	210-230 g	pMCAO for 24 h	Zoletil (50 mg/kg, IM)	Quercetin, 10 mg/kg, IP, at 1 h before MCAO	The same volume of 0.1% DMSO, IP, at 1 h before MCAO	(1) Infarct volume, 24 h after MCAO (4/4)	*P* < 0.05
							(2) Brain water content, 24 h after MCAO (4/4)	*P* < 0.05
							(3) MALDI-TOF analysis for thioredoxin, 24 h after MCAO (4/4)	*P* < 0.05
							(4) Thioredoxin mRNA, 24 h after MCAO (4/4)	*P* < 0.05
							(5) Thioredoxin levels, 24 h after MCAO (4/4)	*P* < 0.05
							(6) Immunofluorescence for thioredoxin, 24 h after MCAO (4/4)	*P* < 0.05
Park et al. 2020b	Male SD rats	210-220 g	pMCAO for 24 h	Zoletil (50 mg/kg, IM)	Quercetin, 10 mg/kg, IP, at 1 h before MCAO	The same volume of 0.1% DMSO, IP, at 1 h before MCAO	(1) NFS (Bederson), 24 h after MCAO (20/20)	*P* < 0.05
							(2) Infarct volume (TTC), 24 h after MCAO (3/3)	*P* < 0.05
							(3) Hippocalcin protein level, 24 h after MCAO (4/4)	*P* < 0.05
							(4) Hippocalcin and NeuN-positive cells, 24 h after MCAO (5/5)	*P* < 0.05
Fan et al. 2020	Male SD rats	220-240 g	tMCAO	Pentobarbital sodium (30 mg/kg, IP)	Quercetin, 25, 50, 100 mg/kg, IG, once daily for 12 days before MCAO	The same volume of normal saline, IG, once daily for 12 days before MCAO	(1) NFS (Longa), 72 h after MCAO (20/20)	*P* < 0.05
							(2) Infarct volume (TTC), 72 h after MCAO (3/3)	*P* < 0.05
							(3) ROS levels, 72 h after MCAO (3/3)	*P* < 0.05
							(4) MDA, 72 h after MCAO (3/3)	*P* < 0.05
							(5) SOD, 72 h after MCAO (3/3)	*P* < 0.05
Park et al. 2021	Male SD rats	220-230 g	pMCAO for 24 h	Zoletil (50 mg/kg, IM)	Quercetin, 10 mg/kg, IP, at 30 min before MCAO	The same volume of 0.1% DMSO, IP, at 30 min before MCAO	(1) NFS (Bederson), 24 h after MCAO (4/4)	*P* < 0.05
							(2) Proteomic for parvalbumin, 24 h after MCAO (4/4)	*P* < 0.05
							(3) Parvalbumin mRNA, 24 h after MCAO (4/4)	*P* < 0.05
							(4) Parvalbumin levels, 24 h after MCAO (4/4)	*P* < 0.05
							(5) Immunostaining of parvalbumin, 24 h after MCAO (4/4)	*P* < 0.05
Yang et al. 2021	Male Wistar rats	280-320 g	tMCAO for 1.5 h	4.5% isoflurane	Quercetin, 10, 30, and 50 mg/kg, IP, at onset of reperfusion	The same volume of DMSO/normal saline, IP, at onset of reperfusion	(1) NFS (mNSS), 24 h after MCAO (7/7)	*P* < 0.001
							(2) Infarct volume (TTC), 24 h after MCAO (7/7)	*P* < 0.001
							(3) BBB permeability (EB), 24 h after MCAO (8/8)	*P* < 0.001
							(4) ROS levels, 24 h after MCAO (6/6)	*P* < 0.05
							(5) ZO-1 expression, 24 h after MCAO (8/8)	*P* < 0.01
							(6) Claudin-5 expression, 24 h after MCAO (8/8)	*P* < 0.01

**Table 2:** Quality assessment of included studies.

Study	A	B	C	D	E	F	G	H	I	J	Total
Ahmad et al. [19]	√				√			√		√	4
Pandey et al. [21]	√	√			√			√			4
Yao et al. [22]	√		√		√						3
Zhang et al. [23]	√	√		√	√			√			5
Shah et al. [24]	√	√	√		√			√	√		6
Park et al. [25]	√	√			√			√			4
Ma et al. [26]	√		√	√	√						4
Park et al. [27]	√	√	√		√						4
Wang et al. [28]	√		√	√				√			4
Park et al. [29]	√	√	√		√			√	√		6
Park et al. [30]	√	√			√			√	√		5
Fan et al. [31]	√	√	√		√				√		5
Park et al. [32]	√	√	√		√			√	√		6
Yang et al. [20]	√		√						√		3

Note: (A) published in a peer-reviewed journal; (B) temperature control; (C) randomization to treatment or control; (D) blinded assessment of outcome; (E) avoidance of intrinsically neuroprotective anesthetics [42]; (F) animal and/or model (aged, diabetic, or hypertensive); (G) sample size calculation; (H) compliance with animal welfare regulations; (I) reporting potential conflicts of interest; (J) injury confirmed via the laser Doppler or perfusion imaging.
